# Three new yeast species of *Vishniacozyma* (*Bulleribasidiaceae*, *Tremellales*) from different habitats

**DOI:** 10.3897/mycokeys.128.175380

**Published:** 2026-02-11

**Authors:** Aleksandra N. Poliakova, Igor A. Cherdantsev, Anna M. Glushakova, Qi-Ming Wang, Dmitry S. Karpov, Nikita B. Polyakov, Andrey I. Solovyev, Vladimir G. Zhukhovitsky, Aleksey V. Kachalkin

**Affiliations:** 1 M.V. Lomonosov Moscow State University, 119234, Moscow, Russia M.V. Lomonosov Moscow State University Moscow Russia; 2 Engelhardt Institute of Molecular Biology, Russian Academy of Sciences, 119991 Moscow, Russia Gamaleya National Research Centre for Epidemiology and Microbiology, Ministry of Health of the Russian Federation Moscow Russia; 3 Center for Molecular and Cellular Biology, Moscow 121205, Russia Russian Medical Academy of Continuing Professional Education (RMANPO), Ministry of Health of the Russian Federation Moscow Russia; 4 G.K. Skryabin Institute of Biochemistry and Physiology of Microorganisms, Federal Research Center Pushchino Scientific Center for Biological Research, Russian Academy of Sciences, 142290, Pushchino, Russia Engelhardt Institute of Molecular Biology, Russian Academy of Sciences Moscow Russia; 5 I.I. Mechnikov Research Institute of Vaccines and Sera, 105064, Moscow, Russia G.K. Skryabin Institute of Biochemistry and Physiology of Microorganisms, Federal Research Center Pushchino Scientific Center for Biological Research, Russian Academy of Sciences Pushchino Russia; 6 School of Life Sciences, Institute of Life Sciences and Green Development, Hebei University, Baoding 071002, Hebei, China Center for Molecular and Cellular Biology Moscow Russia; 7 Gamaleya National Research Centre for Epidemiology and Microbiology, Ministry of Health of the Russian Federation, 123098, Moscow, Russia I.I. Mechnikov Research Institute of Vaccines and Sera Moscow Russia; 8 Russian Medical Academy of Continuing Professional Education (RMANPO), Ministry of Health of the Russian Federation, 125993, Moscow, Russia School of Life Sciences, Institute of Life Sciences and Green Development, Hebei University Baoding China

**Keywords:** *
Basidiomycota
*, cornel, kombucha, MALDI, phylogenetic analysis, *
Scolytus
*, taxonomy, *
Vishniacozyma
*

## Abstract

In this study, three new basidiomycetous yeast species of the genus *Vishniacozyma* are proposed for strains isolated from soil, *Scolytus scolytus* frass, fruits and kombucha tea. A complex analysis of the new isolates together with the described species by genetic, phylogenetic, MALDI-TOF MS profiling and phenotypic characterization revealed significant differences, allowing us to propose three new species: *V. pseudofoliicola***sp. nov**. (holotype KBP Y-7396T, MycoBank no.: MB861627), *V. kombuchae***sp. nov**. (holotype KBP Y-7350T, MycoBank no.: MB861628) and *V. fructicola***sp. nov**. (holotype KBP Y-6599T, MycoBank no.: MB861629). These descriptions contribute to the expansion of knowledge regarding species diversity of the genus *Vishniacozyma*.

## Introduction

*Vishniacozyma* Xin Zhan Liu, F.Y. Bai, M. Groenewald & Boekhout belongs to *Basidiomycota*, *Agaricomycotina*, *Tremellomycetes*, *Tremellomycetidae*, *Tremellales*, and *Bulleribasidiaceae* (http://www.mycobank.org/, accessed 5 August 2025). This genus is proposed for the *dimennae*/victoriae clade including one *Bullera* and six *Cryptococcus* species in 2015 after a phylogenetic revision of some polyphyletic *Tremellomycetes* yeast taxa and named in honor of the American microbiologist Helen S. Vishniac (Liu et al. 2015). Except for *Vishniacozyma indica* and *V. nebularis*, all other members of *Vishniacozyma* are known only by their yeast stage (Crous et al. 2025). The number of species in this genus is constantly increasing. In recent years, *Vishniacozyma* has experienced a rapid influx of proposed new species (Maeng et al. 2022; Zhu et al. 2023; Dlauchy et al. 2024; Jiang et al. 2024; Crous et al. 2025; Golubev 2025; Gungprakhon et al. 2025). There are currently 34 species in the genus *Vishniacozyma* (http://www.mycobank.org/, accessed 15 October 2025), with two additional species proposed in the manuscript by W.I. Golubev (Golubev 2025).

*Vishniacozyma* is a genus of ubiquitous yeasts, whose species are widely distributed in different regions, and they form associations with plants as epiphytes or endophytes and are found in litter and soil as well as in other natural substrates (Liu et al. 2025). Recent studies have demonstrated that the genus *Vishniacozyma* is highly diverse in China, where 20 species are known (Liu et al. 2025). According to the VKM (the All-Russian Collection of Microorganisms, Pushchino, Russia) and KBP (the Yeast collection of the Soil Biology Department at Lomonosov Moscow State University, Moscow, Russia) collections (https://vkm.ru/ and https://depo.msu.ru/, accessed 15 October 2025), only six described species and two proposed species (Golubev 2025) have been found in Russia so far. Consequently, the discovery and descriptions of more *Vishniacozyma* species from this geographic region can be expected.

The aim of this study is to expand the knowledge of this genus by characterizing new strains isolated from various substrates and regions of Russia. The results of their characterization, as well as a comparison with the described *Vishniacozyma* species based on molecular and phenotypic analyses, allow us to propose three new species of the genus. These novel species are named *V. pseudofoliicola*, *V. kombuchae*, and *V. fructicola* spp. nov.

## Materials and methods

### Isolation and phenotypic characterization of the strains

The type strain of the proposed new species, *Vishniacozyma pseudofoliicola*, KBP Y-7396^T^ (former KBP 1600) was isolated in November 1966 by А.V. Kartintsev from a sample of Haplic Gleysols Humic soil (WRB) collected in the Voronezh State Natural Biosphere Reserve named after V.M. Peskov (Voronezh Region). Prior to the current study, this strain was maintained in the working part of VKM collection before being re-deposited in the KBP collection replacing the lost strain KBP 1600. The second strain, KBP Y-7320, was isolated from the frass of *Scolytus scolytus* larvae under the bark on an elm tree collected by A.V. Kachalkin in August 2023 in the settlement of Kosmodem’yanskii (Moscow Region). The type strain of the second new species, *Vishniacozyma kombuchae*, KBP Y-7350^T^ came from a laboratory course on yeast studies. A.N. Poliakova isolated this strain in September 2023 as a minor component of the domestic kombucha tea. The type strain of the third new species, *Vishniacozyma fructicola*, KBP Y-6599^T^ (former KBP YE-0008) was isolated in July 2019 by A.M. Glushakova as part of a study on endophytic yeasts from cornel fruits (*Cornus mas*) collected in the city of Electrougli (Moscow Region).

All yeasts were isolated by plating the prepared suspensions or dilutions to nutrient agar plates. The oldest strain KBP Y-7396^T^ (former KBP 1600) was isolated from a soil suspension using an acidified malt agar from a 1:10 dilution with sterile water. The same dilutions were used with crushed pulp of cornelian cherry, after its surface was sterilized. Other strains were isolated using dry frass material (0.5 g) or aliquot of the kombucha tea (0.5 ml) in sterile water (50 ml) in ratio 1:100. In all cases, for the 2019–2023 isolated strains, after vortexing at 2000 rpm at 15 min on the MultiReax Vortex (Heidolph, Germany), 100 µl of the resulting suspension was used to inoculate plates with GPY agar containing chloramphenicol (Kurtzman et al. 2011). The plates were incubated at 10 °С for 10–14 days in case of frass material, and at 22–25 °С for 7–10 days for kombucha tea and cornelian cherry pulp. The grown yeast colonies were differentiated into macro- and micromorphological types and representative cultures were isolated and identified.

The strains of the new species were characterized morphologically and physiologically using standard methods on solid and liquid media (Kurtzman et al. 2011).

The studied strains were deposited in the Yeast collection of the Soil Biology Department in Lomonosov Moscow State University (KBP – WDCM 1173), in All-Russian Collection of Microorganisms (VKM – WDCM 342), in Industrial Yeasts Collection, Department of Agricultural, Food and Environmental Sciences, University of Perugia (DBVPG – WDCM 180) and in Leibniz Institute DSMZ – German Collection of Microorganisms and Cell Cultures GmbH (DSM – WDCM 274).

### DNA sequencing and phylogenetic analysis

Identification of the yeast strains was based on analysis of the nucleotide sequences of the ITS1-5.8S-ITS2 region (ITS) and the D1/D2 domains of the large subunit (LSU) of the nuclear ribosomal RNA. DNA isolation and PCR were performed according to the previously described procedure (Glushakova and Kachalkin 2017). DNA sequencing was performed using ITS5 (5’ – GGA AGT AAA AGT CGT AAC AAG G) and LR5 (5’ – TCC TGA GGG AAA CTT CG) primers on a 3130xl Genetic Analyzer with the Big Dye Terminator V3.1 Cycle Sequencing Kit (Applied Biosystems, USA) at Evrogen JSC (Moscow, Russia). The nucleotide sequences of the yeast strains were compared with those in public databases using the BLASTn tool from the NCBI (www.ncbi.nlm.nih.gov) and the MycoID tool from MycoBank (www.mycobank.org). The sequences of other genes were investigated for expanded genetic characterization of the new yeast species: the TEF1-α, the largest subunit of RNA polymerase II (RPB1), and the second largest subunit of RNA polymerase II (RPB2). Amplifications and sequencing of the genes were carried out using the following primers: EF-983f and EF-2218r, RPB1-Af and RPB1-Cr, RPB2-7cR and RPB2-5F (details are provided by Kachalkin et al. 2021a). Newly designed primers VF (5’ – TGT GCC ATC CTC ATC ATT GCC A) and VR (5’ – GGG GAT CTG CTC GTG GTG C) were used to amplify the TEF1-α gene in strains KBP Y-7396^T^ and KBP Y-7320 instead of the standard primers. The sequences obtained were deposited in GenBank (Suppl. material 1: table S1).

The nucleotide sequences were assembled using the DNA Baser Sequence Assembler 4 (Heracle BioSoft S.R.L.). To construct the phylogenetic tree of *Vishniacozyma* genus, the sequences of type cultures (list of species according MycoBank) and some other strains (Suppl. material 1: table S1) were aligned using the online version of the MAFFT algorithm with G-INS-I option (Katoh et al. 2019). The tree for the aligned dataset of the ITS and D1/D2 regions was constructed with MEGA6 (Tamura et al. 2013) using the maximum likelihood method based on the Kimura 2-parameter model with gamma-distribution of the invariant sites for 1000 alternative trees for 1009 basepair positions in the final dataset.

### MALDI-TOF mass-spectrometry analysis

In our study, the MALDI-TOF mass-spectrometry analysis (MALDI-TOF MS method) was used to compare the strains. Comparisons were made with the new studied yeast species using the type strains of the closely related species *Vishniacozyma foliicola* AS 2.2471^T^, *Vishniacozyma phoenicis*KBP Y-6564^T^, and strain *Vishniacozyma tephrensis*KBP Y-5908 (GenBank PV635450). All strains were grown under identical conditions: GPYA, 25 °C, 48 hours.

Sample preparation for yeast identification by the MALDI-TOF MS method was carried out according to Sauer et al. (2008) with minor modifications. The biomass of microorganisms was collected with a 1 μl plastic microbiological loop and resuspended in 300 µl of deionized water. We added 900 µl of 96% ethanol to the suspension. The resulting mixture was mixed thoroughly and centrifuged at 18894 g for 2 min. Then we added 5 to 40 µl of 70% formic acid to the air-dried precipitate (depending on the volume of the precipitate) and an equal volume of acetonitrile. The resulting mixture was centrifuged at 18894 g for 2 min. The supernatant containing the protein extract was used in mass spectrometric analyses.

A 384-well steel target plate (Bruker Daltonics, Germany) was covered with 1 µl of supernatant and dried at room temperature. We applied 1 μl of matrix solution – saturated solution of α-cyano-4-hydroxycinnamic acid (25 mg/mL) (Sigma Aldrich, USA) containing 50% acetonitrile and 2.5% trifluoroacetic acid (Panreac, USA) – on the surface of the dried extract, which was also dried at room temperature. Mass spectrometric analyses were performed on an UltrafleXtreme mass spectrometer (Bruker Daltonics, Germany) equipped with a Nd:Yag laser (355 nm) in linear mode. The positively charged ions ranged from 2000 to 20000 Th with the following ion source settings: voltage at IS1 20 kV, at IS2 19 kV, at lenses (parameter “Lens”) 4.5 kV, detector supply voltage 2885 V, gain 12.6.

Spectra were taken in automatic mode using the Flex Control program (v.3.4, build 135). The points of laser firing on the target were chosen randomly. 1200 spectra from 200 firing points at a laser frequency of 2 kHz.

Spectral libraries of each sample were obtained by analyzing biological triplicates in eight technical repeats. Calibration standard and positive control was *Escherichia coli* DH5α protein extract with additional proteins (RNase A [M^+^H] ^+^ 13683.2 Da, myoglobin [M^+^H] ^+^ 16952.3 Da) (cat. no. 255343, Bruker Daltonics, Germany).

The obtained spectra were processed with the MALDI Biotyper Compass Explorer 4.1 software package (Bruker Daltonics, Germany) using the Biotyper Preprocessing Standard Method. The processed spectra of the samples were compared with the reference database of characteristic spectral profiles including 12641 records. The results of the characteristic profile search were expressed as the logarithm of the values. Values below 1.699 corresponded to an unreliable identification of genus; 1.700–1.999 corresponded to a reliable identification of genus and possibly species; 2.000–2.299 corresponded to a reliable identification of genus and with a high probability of species and finally values of 2.300–3.000 corresponded to a reliable identification of species. These criteria have previously been successfully used to identify clinically important yeasts (Marklein et al. 2009; Stevenson et al. 2010).

The MALDIquant package (v1.22.3) (Gibb and Strimmer 2012) was used to process additional mass spectra within the R software environment. Spectral preprocessing included square root intensity transformation, 21-point Savitzky-Golay-Filter (Savitzky and Golay 1964) to smooth the spectra, correction the baseline using the SNIP algorithm with 100 iterations (Ryan et al. 1988) and the Total-Ion-Current-Calibration (TIC) for normalization.

## Results and discussion

### Occurrence and ecology

The strains of the proposed novel species, *Vishniacozyma pseudofoliicola*, KBP Y-7396^T^ (former KBP 1600) and KBP Y-7320, were isolated from different habitats (soil and frass of xylophilous insects) in the Voronezh and Moscow Regions of Russia in 1966 and 2023, respectively. Unfortunately, information on other yeasts found in soil in 1966 is currently not available. The strain KBP Y-7320 was isolated from a sample of *Scolytus scolytus* frass under the rotting bark of an elm tree, together with other yeasts (*Aureobasidium pullulans*, *Candida peoriensis*, *Holtermanniella takashimae*, *Rhodosporidiobolus colostri*, *Vishniacozyma carnescens*, and *Wickerhamomyces* sp. (GenBank OR582615)) and the plant pathogen *Ophiostoma novo-ulmi*. The ITS regions of these isolated fungi were almost identical (99.8–100% similar) to the type strains of those species, with the exception of *Wickerhamomyces* sp. The new *Vishniacozyma* species accounted for 23.8% of the total yeast abundance at nearly 2.5×10^6^ CFU/g.

The single strain of the novel species, *Vishniacozyma kombuchae*, KBP Y-7350^T^ isolated as a minor component or contaminant of the domestic kombucha tea together with *Metschnikowia pulcherrima*. Perhaps our method was not suitable for the isolation of other typical kombucha yeasts (Teoh et al. 2004) because the cells were exposed to osmotic shock during the preparation of the suspension.

The type strain of the third new species, *Vishniacozyma fructicola*, was isolated as a minor component in a study on endophytic yeasts from cornel fruits (Kachalkin et al. 2021b). In addition to the new species, *Aureobasidium pullulans* and *Leucosporidium scottii* were also found in pulp with a total yeast abundance of 6.3×10^2^ CFU/g.

The detection of *Vishniacozyma* in soils and litter is a common finding that has been repeatedly reported in other studies, including DNA metabarcoding (Glushakova et al. 2017; Mašínová et al. 2017; Yurkov 2018). Type strains of nine species were isolated from soils (Suppl. material 1: table S1). However, the typical habitat of species of this genus is more often associated with plants (Yurkov 2018). Many *Vishniacozyma* yeasts are also found in association with plants (Suppl. material 1: table S1), including diseased *Ulmus* trees (Marčiulynas et al. 2022; Macaya-Sanz et al. 2023), and with some insects (Oono et al. 2024), including xylophilous insects (Morales-Rodríguez et al. 2021; Davydenko et al. 2025). Therefore, insects may act as vectors in the spread of new species *Vishniacozyma* among different substrates.

### Genetic delineation and phylogenetic placement

**Figure 1.**
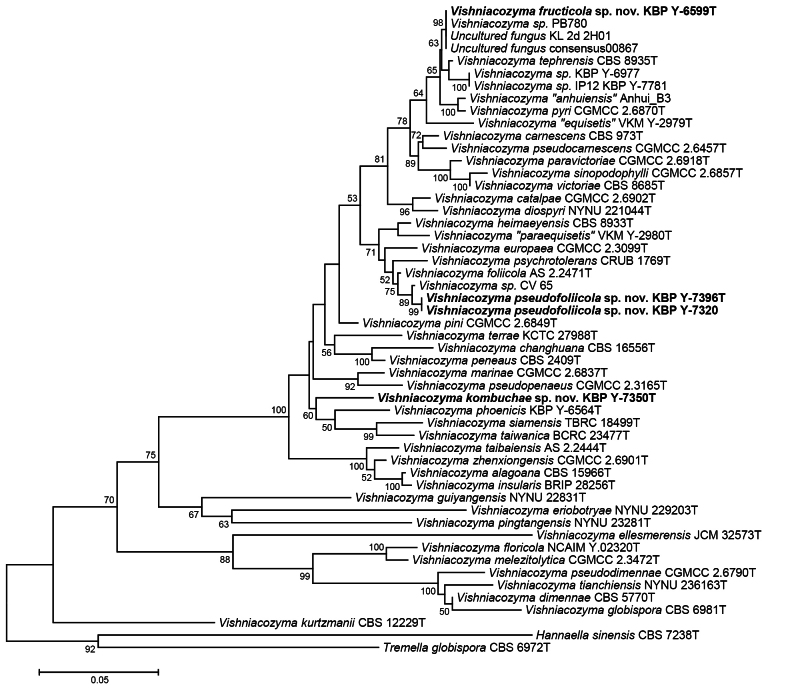
Maximum likelihood (ML) phylogenetic tree of *Vishniacozyma* species and strains (listed in Suppl. material 1: table S1) inferred using the combined sequences of ITS and LSU regions. The bootstrap values (over 50%) for 1000 replicates are indicated at the nodes. Scale bar represents 0.05 substitutions per nucleotide position. *Hannaella sinensis* and *Tremella globispora* were used as the outgroup, according Dlauchy et al. (2024).

The phylogenetic placement of the novel species inferred by the maximum likelihood method by concatenated alignment of the ITS region and the LSU D1/D2 domain is shown in Fig. 1. The new species were placed in clades that were well to moderately (≥60%) statistically supported, alongside the following closely related species: *V. foliicola*, *V. phoenicis* and *V. tephrensis*.

Strains KBP Y-7396^T^ and KBP Y-7320 are conspecific (identity = 100%) according to the ITS and D1/D2 LSU rRNA gene sequences. Our BLASTn search and phylogenetic tree revealed that they are most closely related to *V. foliicola* AS 2.2471^T^ (Fig. 1), but they differ from that species by more than 1.8% (9 substitutions) along the 526 basepairs long alignment in the ITS region and by 0.3% (2 substitutions) along the 586 basepairs in the D1/D2 LSU. Such genetic similarity data do not provide unambiguous information about whether the strains we studied belong to a new species or not. Previous studies have derived the following thresholds for *Basidiomycota* yeast species: 98.61% using ITS barcodes and 99.51% using LSU, but near 3% yeast species were indistinguishable by both loci (Vu et al. 2016). Therefore, we used a complex approach involving the analysis of secondary barcode genes, MALDI-TOF MS profiling, and phenotypic characterization. The nucleotide sequence of the TEF1-α gene shows 8.3% differences (23 substitutions and 25 indels) between KBP Y-7396^T^ and *V. foliicola* AS 2.2471^T^ along the 599 basepairs, while there are no differences between the KBP Y-7396^T^ and KBP Y-7320 strains. Examination of the RNA polymerase II genes revealed the following differences between *V. foliicola* AS 2.2471^T^ and KBP Y-7396^T^: 11.6% for RPB1 and 12.4% for RPB2; by comparison, the differences between the strains KBP Y-7396^T^ and KBP Y-7320 were 0.4% and 0%. Similarly, a difference of 12.4% is observed when comparing the type strains of closely related species, such as *V. sinopodophylli* and *V. victoriae*, by RPB2 gene. These differences in protein-coding genes suggest that the proposed new species *V. pseudofoliicola* and *V. foliicola* have diverged genetically. The strain *Vishniacozyma* sp. CV_65 (GenBank HG994936) from the USA, which is close to the species, requires additional studies to assign it to the proposed new species or to *V. foliicola*, or a new species.

The proposed new species *V. kombuchae* seems to be phylogenetically close to *V. phoenicis*, *V. siamensis* and *V. taiwanenica*, although without high bootstrap support (Fig. 1). According to an NCBI GenBank search, the strain KBP Y-7350^T^ differs from the *V. phoenicis*KBP Y-6564^T^ by 5.1% (9 substitutions and 14 indels) along the 528 basepairs in the ITS region and by 1% (5 substitutions) along the 516 basepairs in the D1/D2 LSU. The nucleotide sequence of the protein-coding genes shows 17% differences for RPB1 and 8.4% for TEF1-α from *V. phoenicis*. The differences obtained for the ribosomal and RPB1 genes, as well as for TEF1-α genes (with the identical value obtained for both *V. pseudofoliicola* and *V. foliicola*), demonstrate genetic differences between the proposed new species *V. kombuchae* and *V. phoenicis*.

The third proposed yeast species, *V. fructicola*, is phylogenetically related to *V. tephrensis* and the strains KBP Y-6977 and KBP Y-7781, which may also be a new species that requires further research (Fig. 1). There also exist in NCBI database a number of conspecific nucleotide sequences of uncultured fungi KL_2d_2H01 (GenBank JF495251) from beech litter in Austria and Consensus00867 (GenBank OU939573) from soil in Sweden, and also one isolate PB780 (GenBank KX078413) from fermenting wine in Italy. The strain KBP Y-6599^T^ differs by 2% (10 substitutions) along the 497 basepairs in the ITS region and by 0.2% (one substitution) along the 594 basepairs in the D1/D2 LSU from the *V. tephrensis* CBS 8935^T^. The levels of difference between strains CBS 8935^T^ and KBP Y-6599^T^ for the other genes we analyzed are as follows: 16.7% for RPB1, 12.3% for RPB2, and 12.5% for TEF1-α. Thus, despite almost no differences in LSU, the obtained genetic differences between the strains support distinguishing *V. fructicola* as a new species.

We would also like to highlight that more and more new yeast species belonging to both the ascomycetes and the basidiomycetes are being discovered whose ribosomal genes are identical to those of known species or differ only slightly (i.e. Touchette et al. 2022; Peña et al. 2024). These genes have traditionally been used to distinguish yeast species (Vu et al. 2016; Boekhout et al. 2021). However, this situation is gradually changing and can already be observed in some groups of micro- and macromycetes, where ribosomal genes are not always used to identify and classify species (e.g., Lücking et al. 2020; Crous et al. 2021).

### MALDI-TOF MS analysis

**Figure 2.**
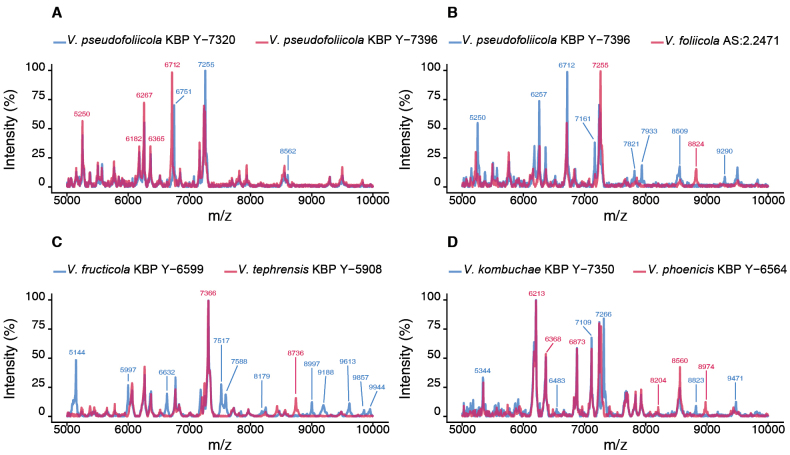
Comparison of MALDI-TOF mass spectra of *Vishniacozyma* species at m/z 5000–10000: **A**. *V. pseudofoliicola* sp. nov. KBP Y-7396^T^ and KBP Y-7320; **B**. *V. pseudofoliicola* sp. nov. KBP Y-7396^T^ and *V. foliicola* AS 2.2471^T^; **C**. *V. fructicola* sp. nov. KBP: Y-6599^T^ and *V. tephrensis*KBP Y-5908; **D**. *V. kombuchae* sp. nov. KBP Y-7350^T^ and *V. phoenicis*KBP Y-6564^T^.

MALDI-TOF MS was used to differentiate the studied Vishniacozyma strains from type strains and reference cultures. The MALDI-TOF mass spectra of the *V. foliicola* AS 2.2471^T^, *V. tephrensis*KBP Y-5908 (GenBank PV635450), *V. phoenicis*KBP Y-6564^T^ were used to construct an in-house database. The results for the studied strains, compared with the reference strains, were in the range 2.00–2.28, which reliably indicates the single genus level. The strain *V. fructicola*KBP Y-6599^T^ matches to *V. tephrensis*KBP Y-5908 with the best scores 2.00–2.08. The similarities in protein spectra between *V. kombuchae*KBP Y-7350^T^ and *V. phoenicis*KBP Y-6564^T^ showed the best match 2.20. *V. pseudofoliicola*KBP Y-7396^T^ and KBP Y-7320 strains were identified as *V. foliicola* AS 2.2471^T^ with best matches scores of 2.28 and 2.23, respectively. The similarity score of 2.77 between strains KBP Y-7396^T^ and KBP Y-7320 indicates that they are the same species. The differences between the mass spectra of the species pairs are shown in Fig. 2.

In general, the variance in the mass spectra of KBP Y-7396^T^ and KBP Y-7320 of *V. pseudofoliicola* is barely noticeable. Peaks in the m/z range of 6715–6755 showed a slight shift, and a more intense peak at m/z 8562 was present in strain KBP: Y-7320 compared to KBP: Y-7396^T^. However, the protein profiles of the type strains of the other proposed species differ from those of the reference strains. *V. pseudofoliicola*KBP Y-7396^T^ and *V. foliicola* AS 2.2471^T^ exhibit variability at 5250 and 7161 m/z, as well as differences in unique peaks. The protein profile of *V. pseudofoliicola*KBP Y-7396^T^ has singular peaks at 7821, 7932 and 9290 m/z, but not at 8824 m/z as seen in *V. foliicola* AS 2.2471^T^. In turn, *V. kombuchae*KBP Y-7350^T^ can be differentiated from the reference strain, *V. phoenicis*KBP Y-6564^T^, by the presence of peaks in the 6483 and 8823 m/z and absence 8204 and 8974 m/z peaks, and a small shift is observed in the range around 7200 m/z. And *V. fructicola*KBP Y-6599^T^ can be distinguished from used for comparisons *V. tephrensis*KBP Y-5908 by peaks in the 5144, 6632, 7517–7588, 8179, 8997, 9187, 9613, 9857–9944 m/z. The protein profile of *V. tephrensis*KBP Y-5908 has unique 8736 m/z peak that is missing in *V. fructicola*KBP Y-6599^T^.

Therefore, the genetic features of the studied strains and the MALDI-TOF MS data provide increasing evidence for the proposal of three new species of *Vishniacozyma*.

### Phenotypic characters

**Figure 3.**
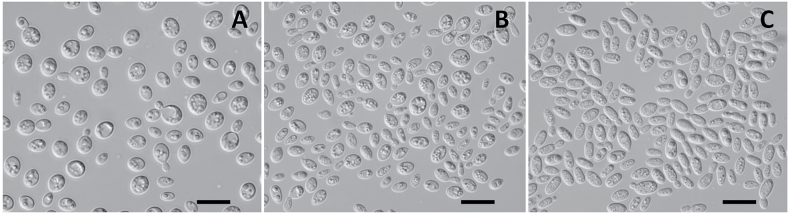
The budding cells of **A**. *V. pseudofoliicola* sp. nov. KBP Y-7396^T^; **B**. *V. kombuchae* sp. nov. KBP Y-7350^T^ and **C**. *V. fructicola* sp. nov. KBP: Y-6599^T^ grown on MEA for 7 days at 25 °C. Scale bars: 10 μm.

The cells of *V. pseudofoliicola* sp. nov. (Fig. 3A) are predominantly globose and ovoid, as opposed to the ovoidal and ellipsoidal to elongate cells of *V. foliicola* (Wang et al. 2011). This new yeast species can be differentiated from *V. foliicola* physiologically due to its inability to assimilate inulin, D-glucosamine, galactitol, D-mannitol, glucitol, salicin and ethylamine (as a nitrogen source), but can grow on media containing 50% glucose and with 0.01% cycloheximide (Suppl. material 2: table S2).

The cell of *V. kombuchae* sp. nov. (Fig. 3B) is similar to *V. phoenicis* (Crous et al. 2020). However, *V. kombuchae* sp. nov. has a number of notable physiological differences. These include an inability to assimilate soluble starch, ethanol, glycerol, succinic acid, citric acid, and some nitrogen sources (potassium nitrate and creatine), and an inability to grow on media without vitamins, but it can assimilate D-glucosamine as a carbon source (Suppl. material 2: table S2).

*V. tephrensis* have ovoid cells (Vishniac 2002) whereas the cells of *V. fructicola* sp. nov. are larger and mainly ellipsoidal to elongate (Fig. 3C). *V. tephrensis* can be distinguished from the *V. fructicola* sp. nov. by the following physiological features: an ability to assimilate soluble starch, inulin, galactitol, and *V. fructicola* sp. nov. can also assimilate D-mannitol, sorbitol, DL-lactic acid, and some nitrogen sources (potassium nitrate and creatinine), and by growing on media with 50% Glucose and with 10% NaCl, and without vitamins (Suppl. material 2: table S2).

The newly proposed species differ from their closest relatives by eight to eleven physiological characteristics. In the case of *V. pseudofoliicola* sp. nov. and *V. fructicola* sp. nov., there are also some differences in micromorphology. This indicates that new species can be distinguished from other species by their physiological characteristics and some morphological features, i.e. a phenotypic concept for yeast species.

Based on the above data, which must be considered together (i.e. genetic delineation, MALDI-TOF MS analysis and phenotypic characters), we propose the following three new yeast species: *V. pseudofoliicola*, *V. kombuchae* and *V. fructicola* spp. nov. These descriptions contribute to the expansion of knowledge on species diversity. Three new species of the genus *Vishniacozyma* have been added to the list of yeasts from Russia.

### Taxonomic novelties

#### Vishniacozyma pseudofoliicola

Taxon classificationFungiTremellalesBulleribasidiaceae

A.N. Poliakova, A.V. Kachalkin sp. nov.

45E537EE-8431-5A97-9056-011A865E5596

MB861627

Fig. 3A

##### Etymology.

The name *Vishniacozyma pseudofoliicola* (pseu.do.fo.li.i’co.la. N.L. fem. adj.) refers to a similarity with *V. foliicola*.

##### Description.

When growing on 5% malt extract agar (MEA) after 7 days at 25 °C, the streak culture is butyrous, glistening, smooth and flat, with an entire margin, ivory to faint beige in color with a slightly lighter color at the margin. The color darkens with age, the lighter margin is retained. On potato dextrose agar (PDA) after 7 days at 25 °C, the streak culture is same as on MEA, but slightly raised and with uniform color. Cells are globose and ovoid (2.5–6.6 × 3.6–7.5 µm) and occur singly or in pairs (Fig. 3A). Budding is multilateral. Ballistoconidia are not produced. Mycelium, pseudomycelium and sexual structures (mating test) are not observed on MEA, PDA, corn meal agar (CMA) and V8 agar at 10 °C and 25 °C over a period of two months.

Glucose is not fermented. Positive growth on glucose, galactose, sucrose, maltose, cellobiose, trehalose, lactose, melibiose, raffinose, melizitose, D-xylose, L-arabinose, D-arabinose (weak and slow), D-ribose (weak and variable), L-rhamnose, erythritol, ribitol (weak and variable), myo-inositol, methyl-α-d-glucoside, D-glucuronic acid (positive or slow), DL-lactic acid (weak), succinic acid (positive or slow), citric acid (slow or weak), 2-Keto-D-gluconate, 5-Keto-D-gluconate, arbutin. No growth on sorbose, inulin, soluble starch, D-glucosamine, methanol, ethanol, glycerol, galactitol, D-mannitol, glucitol, salicin, and hexadecane. Ammonium sulphate, lysine and D-glucosamine are assimilated as sole nitrogen sources, but potassium nitrate, ethylamine hydrochloride and creatine are not assimilated. Growth in the vitamin-free medium is positive. Extracellular starch-like compounds are produced. Weak growth may occur (variable test) in 10 % (w/v) sodium chloride with 5 % (w/v) glucose medium, but no at 16% (w/v) sodium chloride. Weak growth may occur in the presence of 0.01% cycloheximide, but growth is absent with 0.1% cycloheximide. Growth on 50 % (w/v) glucose-yeast extract agar is positive. Urea is hydrolyzed, and the color reaction with diazonium blue B is positive. The maximal growth temperature is 28 °C.

##### Typus.

Russia • Voronezh Region: Voronezh State Natural Biosphere Reserve by V.M. Peskov, from a Haplic Gleysols Humic soil, November 1966, А.V. Kartintsev, KBP 1600 (holotype KBP Y-7396^T^ preserved as a metabolically inactive state, cultures ex-type DBVPG 8080 and VKM Y-3715).

##### Additional strain examined.

Russia • Moscow Region: settlement of Kosmodem’yanski, from the frass of *Scolytus scolytus* larvae under the bark of elm tree, August 2023, A.V. Kachalkin, KBP Y-7320 (=VKM Y-4103).

##### GenBank accession numbers.

Holotype KBP Y-7396^T^ (ITS and D1/D2 LSU: OP602955; RPB1: PV029737; RPB2: PV029733; TEF1-α: PV029735); additional strain KBP Y-7320 (ITS and D1/D2 LSU: OR623241; RPB1: PV029738; RPB2: PV029734; TEF1-α: PV029736).

#### Vishniacozyma kombuchae

Taxon classificationFungiTremellalesBulleribasidiaceae

A.N. Poliakova, A.V. Kachalkin sp. nov.

8E483CFE-AFC8-5447-8379-5D410FDA1021

MB861628

Fig. 3B

##### Etymology.

The name *Vishniacozyma kombuchae* (*kom.bu'chae*. N.L. gen. fem. n.) refers to the isolation of the type strain from the kombucha tea.

##### Description.

When growing on 5% malt extract agar (MEA) and on potato dextrose agar (PDA) after 7 days at 25 °C, the streak culture is mucoid, glistening, smooth and flat, with an entire margin, faint beige in color. The color darkens with age. Cells are globose, ovoid and ellipsoidal (1.6–6.0 × 3.0–7.5 µm) and occur singly or in pairs (Fig. 3B). Budding is polar. Ballistoconidia are not produced. Mycelium, pseudomycelium and sexual structures are not observed on MEA, PDA, corn meal agar (CMA) and V8 agar at 10 °C and 25 °C over a period of two months.

Glucose is not fermented. Positive growth on glucose, galactose, sorbose (slow), sucrose, maltose, cellobiose, trehalose, lactose, melibiose, raffinose, melizitose, D-xylose, L-arabinose, D-arabinose, D-ribose, L-rhamnose, D-glucosamine (slow), erythritol, ribitol (weak), galactitol, D-mannitol, glucitol, myo-inositol, methyl-α-d-glucoside, salicin (weak), DL-lactic acid (weak), D-glucuronic acid, 5-Keto-D-gluconate and arbutin (weak). No growth on inulin, soluble starch, methanol, ethanol, glycerol, succinic acid, citric acid, and hexadecane. Ammonium sulphate, lysine and D-glucosamine are assimilated as sole nitrogen sources, but potassium nitrate, ethylamine hydrochloride and creatine are not assimilated. Growth in the vitamin-free medium is negative. Extracellular starch-like compounds are produced. Weak growth in 10 % (w/v) sodium chloride with 5 % (w/v) glucose medium, but no at 16% (w/v) sodium chloride. Weak growth may occur in the presence of 0.01% cycloheximide, but growth is absent with 0.1% cycloheximide. Growth on 50 % (w/v) glucose-yeast extract agar is weak and variable. Urea is hydrolysed, and the color reaction with diazonium blue B is positive. The maximal growth temperature is 30 °C.

##### Typus.

Russia • Moscow: from home-made kombucha tea, September 2024, A.N. Poliakova, KBP Y-7350 (holotype KBP Y-7350^T^ preserved as a metabolically inactive state, cultures ex-type DBVPG 8078 and VKM Y-4117).

##### GenBank accession numbers.

Holotype KBP Y-7350^T^ (ITS and D1/D2 LSU: PP294695; RPB1: PV029730; RPB2: PV029731; TEF1-α: PV029732).

#### Vishniacozyma fructicola

Taxon classificationFungiTremellalesBulleribasidiaceae

A.N. Poliakova, A.M. Glushakova, A.V. Kachalkin sp. nov.

AE9764E2-4323-5C36-82DF-3B8D043DC930

MB861629

Fig. 3C

##### Etymology.

The name *Vishniacozyma fructicola* (*fruc.ti'co.la*. N.L. masc. n.) refers to isolation of the type strain from the fruit of cornel.

##### Description.

When growing on 5% malt extract agar (MEA) and on potato dextrose agar (PDA) after 7 days at 25 °C, the streak culture is butyrous, glistening, smooth and flat, with an entire margin, faint beige to beige in color with a slightly lighter color at the margin. Cells are ovoid, ellipsoidal to elongate (1.8–4.2 × 3.8–7.0 µm) and occur singly or in pairs (Fig. 3C). Budding is mainly polar but can also be bipolar or multilateral. Ballistoconidia are not produced. Mycelium, pseudomycelium and sexual structures are not observed on MEA, PDA, corn meal agar (CMA) and V8 agar at 10 °C and 25 °C over a period of two months.

Glucose is not fermented. Positive growth on glucose, sorbose (weak), sucrose, maltose, cellobiose, trehalose, lactose, melibiose, raffinose, melizitose, D-xylose, L-arabinose, D-arabinose, D-ribose, L-rhamnose, D-glucosamine, glycerol, erythritol, ribitol, D-mannitol, glucitol, myo-inositol, methyl-α-d-glucoside, salicin, D-glucuronic acid (weak), DL-lactic acid (weak), succinic acid, 2-Keto-D-gluconate, 5-Keto-D-gluconate, and arbutin. No growth on inulin, soluble starch, methanol, ethanol, galactitol, citric acid. Ammonium sulphate, potassium nitrate, lysine, creatine, creatinine, and D-glucosamine are assimilated as sole nitrogen sources, but ethylamine hydrochloride and cadaverine are not assimilated. Growth in the vitamin-free medium is positive. Extracellular starch-like compounds are produced. Growth in 10 % (w/v) sodium chloride with 5 % (w/v) glucose medium, but no at 16% (w/v) sodium chloride. Weak growth may occur in the presence of 0.01% cycloheximide, but growth is absent with 0.1% cycloheximide. Growth on 50 % (w/v) glucose-yeast extract agar is positive. Urea is hydrolysed, and the color reaction with diazonium blue B is positive. The maximal growth temperature is 29 °C.

##### Typus.

Russia • Moscow Region: city of Electrougli, from cornel fruits, July 2019, A.M. Glushakova, KBP YE-0008 (holotype KBP Y-6599^T^ preserved as a metabolically inactive state, cultures ex-type DSM 110869 and VKM Y-3606).

##### GenBank accession numbers.

Holotype KBP Y-6599^T^ (ITS and D1/D2 LSU: MT013023; RPB1: PV061054; RPB2: PV061055; TEF1-α: PV061056).

##### Note.

*Vishniacozyma fructicola* has been placed in clade (Fig. 1) includes isolate PB780 (GenBank KX078413) from fermenting wine in Italy, which is the same species.

## Supplementary Material

XML Treatment for Vishniacozyma pseudofoliicola

XML Treatment for Vishniacozyma kombuchae

XML Treatment for Vishniacozyma fructicola

## Additional information

### Use of AI

No use of AI was reported.

### Author ORCIDs

Aleksandra N. Poliakova https://orcid.org/0009-0004-0734-0056

Igor A. Cherdantsev https://orcid.org/0009-0008-1639-0660

Anna M. Glushakova https://orcid.org/0000-0002-4116-4445

Qi-Ming Wang https://orcid.org/0000-0002-2180-8135

Dmitry S. Karpov https://orcid.org/0000-0001-5203-0787

Nikita B. Polyakov https://orcid.org/0000-0002-1647-5815

Andrey I. Solovyev https://orcid.org/0000-0002-7389-6157

Vladimir G. Zhukhovitsky https://orcid.org/0000-0002-4653-2446

Aleksey V. Kachalkin https://orcid.org/0000-0002-4494-2468

### Data availability

The datasets used and/or analyzed during the current study are available from the corresponding author upon reasonable request.

## Supplementary materials

Supplementary material 1 Strains of the genus *Vishniacozyma* used in the phylogenetic analysis with their GenBank accession numbers Data type docx https://binary.pensoft.net/file/1538362This dataset is made available under the Open Database License (http://opendatacommons.org/licenses/odbl/1.0/). The Open Database License (ODbL) is a license agreement intended to allow users to freely share, modify, and use this Dataset while maintaining this same freedom for others, provided that the original source and author(s) are credited. Aleksandra N. Poliakova, Igor A. Cherdantsev, Anna M. Glushakova, Qi-Ming Wang, Dmitry S. Karpov, Nikita B. Polyakov, Andrey I. Solovyev, Vladimir G. Zhukhovitsky, Aleksey V. Kachalkin

Supplementary material 2 Phenotypic properties of the novel *Vishniacozyma* species and phylogenetically closely related species Data type docx https://binary.pensoft.net/file/1538363This dataset is made available under the Open Database License (http://opendatacommons.org/licenses/odbl/1.0/). The Open Database License (ODbL) is a license agreement intended to allow users to freely share, modify, and use this Dataset while maintaining this same freedom for others, provided that the original source and author(s) are credited. Aleksandra N. Poliakova, Igor A. Cherdantsev, Anna M. Glushakova, Qi-Ming Wang, Dmitry S. Karpov, Nikita B. Polyakov, Andrey I. Solovyev, Vladimir G. Zhukhovitsky, Aleksey V. Kachalkin
