# Supplementary material for: Three new yeast species of Vishniacozyma (Bulleribasidiaceae, Tremellales) from different habitats
Source: MycoKeys. 2026 Feb 11;128:231–48. doi: 10.3897/mycokeys.128.175380 (PMC12917494; doi:10.3897/mycokeys.128.175380)
Supplement: Supplementary material 1 — Strains of the genus Vishniacozyma used in the phylogenetic analysis with their GenBank accession numbers [file mycokeys-128-231-s001.docx]

**Table S1.** Strains of the genus *Vishniacozyma* used in the phylogenetic analysis with their GenBank accession numbers (sequences generated in this study are indicated in bold font).

| **Species** | **Strain ID** | **Isolation source** | **Location** |  |  |  |  |  | **References** |
| --- | --- | --- | --- | --- | --- | --- | --- | --- | --- |
|  |  |  |  | **ITS** | **D1/D2** | **RPB1** | **RPB2** | **TEF1a** |  |
| ***Vishniacozyma pseudofoliicola* sp.nov*.*** | **KBP Y-7396**^T^ | Soil | Voronezh Region, Russia | **OP602955** | **OP602955** | **PV029737** | **PV029733** | **PV029735** | **This study** |
| ***Vishniacozyma pseudofoliicola* sp.nov*.*** | **KBP Y-7320** | Frass of *Scolytus scolytus* | Moscow Region, Russia | **OR623241** | **OR623241** | **PV029738** | **PV029734** | **PV029736** | **This study** |
| ***Vishniacozyma kombuchae* sp.nov*.*** | **KBP Y-7350**^T^ | Kombucha tea | Moscow, Russia | **PP294695** | **PP294695** | **PV029730** | **PV029731** | **PV029732** | **This study** |
| ***Vishniacozyma fructicola* sp.nov*.*** | **KBP Y-6599**^T^ | Cornel fruits | Moscow, Russia | **MT013023** | **MT013023** | **PV061054** | **PV061055** | **PV061056** | **This study** |
| ***Vishniacozyma* sp*.*** | **KBP Y-6977** | Frass of *Agrilus planipennis* | Moscow, Russia | **OP941489** | **OP941489** |  |  |  | **This study** |
| ***Vishniacozyma* sp. IP12** | **KBP Y-7781** | Soil | Moscow, Russia | **PV000763** | **PV000763** |  |  |  | **This study** |
| *Vishniacozyma* sp*. CV_65* |  | Olive fly | California, USA | HG994936 | HG994936 |  |  |  | NCBI data |
| *Uncultured fungus,* consensus00867 |  | Soil | Sweden | OU939573 | OU939573 |  |  |  | NCBI data |
| *Uncultured fungus,* KL_2d_2H01 |  | Beech litter | Klausenleopoldsdorf, Austria | JF495251 | JF495251 |  |  |  | NCBI data |
| *Vishniacozyma* sp*.* PB780 |  | Fermenting wine | Italy | KX078413 | KX078413 |  |  |  | NCBI data |
| *Vishniacozyma "anhuiensis"* | Anhui_B3 | Fruits | China | MN450784 | MN450784 |  |  |  | NCBI data |
| *Vishniacozyma "equisetis"* | VKM Y-2979^T^ | *Equisetum sylvaticum* | Moscow Region, Russia | HM749321 | HM749316 |  |  |  | Golubev 2025 |
| *Vishniacozyma "paraequisetis"* | VKM Y-2980^T^ | *Equisetum sylvaticum* | Moscow Region, Russia | HM749319 | HM749318 |  |  |  | Golubev 2025 |
| *Vishniacozyma alagoana* | CBS 15966^T^ | Leaf of unidentified *Bromeliaceae* | Brazil | MH885328 | MH909005 |  |  |  | Félix et al. 2020 |
| *Vishniacozyma carnescens* | CBS 973^T^ | Muscatel grape | Italy | KY105817 | AB035054 | KF036354 | KF036767 | KF037039 | Takashima et al. 2003; Liu et al. 2015 |
| *Vishniacozyma catalpae* | CGMCC 2.6902^T^ | Leaf of *Catalpa ovata* | Yunnan province, China | OP470302 | OP470206 | OP784874 | OP771485 | OP853471 | Jiang et al. 2024 |
| *Vishniacozyma changhuana* | CBS 16556^T^ | Leaves of *Avicennia marina* | Taiwan | MT906456 | MT906468 |  |  |  | Chang et al. 2021 |
| *Vishniacozyma dimennae* | CBS 5770^T^ | Pasture plants | New Zealand | AF410473 | AF075489 | KF036364 | KF036776 | KF037049 | Fell and Phaff 1967; Liu et al. 2015 |
| *Vishniacozyma diospyri* | NYNU 221044^T^ | Phylloplane of *Diospyros lotus* | Henan province, China | OP954624 | OP954569 |  |  |  | Liu et al. 2025 |
| *Vishniacozyma ellesmerensis* | JCM 32573^T^ | Sediments and soil at the front of a retreating glacier | Canada | LC335796 | LC335796 |  |  |  | Tsuji et al. 2019 |
| *Vishniacozyma eriobotryae* | NYNU 229203^T^ | Phylloplane of *Eriobotrya japonica* | Guizhou province, China | OP566897 | OP566895 |  |  |  | Liu et al. 2025 |
| *Vishniacozyma europaea* | CGMCC 2.3099^T^ | Phylloplane | Germany | MK050335 | MK050335 | MK849148 |  | MK849018 | Li et al. 2020 |
| *Vishniacozyma floricola* | NCAIM Y.02320^T^ | From flowers of different plant species | Hungary | PP337022 | PP261370 |  |  |  | Dlauchy et al. 2024 |
| *Vishniacozyma foliicola* | AS 2.2471^T^ | Plant leaves | Hubei province, China | AY557600 | AY557599 | **PV013602** | **PV013603** | **PV013601** | Wang et al. 2011; **This study** |
| *Vishniacozyma globispora* | CBS 6981^T^ | Surface of rotten wood | Canada | AF444407 | AF075509 | KF036323 | KF036736 | KF037007 | Liu et al. 2015 |
| *Vishniacozyma guiyangensis* | NYNU 22831^T^ | Phylloplane of *Distylium racemosum* | Guizhou province, China | OP566869 | OP566870 |  |  |  | Liu et al. 2025 |
| *Vishniacozyma heimaeyensis* | CBS 8933^T^ | Soil | Iceland | HQ875391 | DQ000317 | KF036376 | KF036788 | KF037060 | Vishniac 2002; Liu et al. 2015 |
| *Vishniacozyma insularis* | BRIP 28256^T^ | *Banksia* sp. | Queensland, Australia | OK442366 | OP167985 |  |  |  | Tan et al. 2021 |
| *Vishniacozyma kurtzmanii* | CBS 12229^T^ | The surface of maize kernels | Minnesota, USA | MH718303 | MH718303 |  |  |  | Yurkov and Kurtzman 2019 |
| *Vishniacozyma marinae* | CGMCC 2.6837^T^ | Seawater | Hainan, China | OP470294 | OP470198 | OP784870 | OP771477 | OP853469 | Jiang et al. 2024 |
| *Vishniacozyma melezitolytica* | CGMCC 2.3472^T^ | Phylloplane | Hebei province, China | MK050330 | MK050330 | MK849177 | MK849315 | MK849046 | Li et al. 2020 |
| *Vishniacozyma nebularis* | CBS 122283^T^ | Dead branch | Taiwan | - | EU266921 |  |  |  | Kirschner and Chen 2008 |
| *Vishniacozyma paravictoriae* | CGMCC 2.6918^T^ | Soil | Tibet, China | OP470300 | OP470204 | OP853417 | OP771483 | OP853517 | Jiang et al. 2024 |
| *Vishniacozyma peneaus* | CBS 2409^T^ | Surface washing of shrimp | Texas, USA | AB035047 | AB035051 | KF036392 | KF036806 | KF037077 | Takashima et al. 2003; Liu et al. 2015 |
| *Vishniacozyma phoenicis* | KBP Y-6564^T^ | Fruit, dates | Moscow, Russia | MN449981 | MN449981 | LR701187 |  | LR701186 | Crous et al. 2020 |
| *Vishniacozyma pingtangensis* | NYNU 23281^T^ | Phylloplane of *Acer saccharum* | Guizhou province, China | OQ851896 | OQ851894 |  |  |  | Liu et al. 2025 |
| *Vishniacozyma pini* | CGMCC 2.6849^T^ | Bark of *Pinus* sp. | Tibet, China | OP470296 | OP470200 | OP784872 | OP771479 | OP853493 | Jiang et al. 2024 |
| *Vishniacozyma pseudocarnescens* | CGMCC 2.6457^T^ | Marine water | Liaoning province, China | OR077051 | OR077057 | OR103280 | OR103286 | OR120033 | Zhu et al. 2023 |
| *Vishniacozyma pseudodimennae* | CGMCC 2.6790^T^ | Plant tissue | Qinghai province, China | OM417179 | OM417179 |  |  |  | Wei et al. 2022 |
| *Vishniacozyma pseudopenaeus* | CGMCC 2.3165^T^ | Phylloplane | Germany | MK050333 | MK050333 | MK849155 |  | MK849025 | Li et al. 2020 |
| *Vishniacozyma psychrotolerans* | CRUB 1769^T^ | Subglacial ice | Austre lovénbreen glaciers, Norway | AB035049 | JN193445 |  |  |  | De Garcia et al. 2012 |
| *Vishniacozyma pyri* | CGMCC 2.6870^T^ | Leaf of *Catalpa ovata* | Tibet, China | OP470298 | OP470202 | OP853422 | OP771481 | OP853515 | Jiang et al. 2024 |
| *Vishniacozyma siamensis* | TBRC 18499^T^ | Leaf surfaces of mangrove plants | Thailand | LC786857 | LC797015 |  |  |  | Gungprakhon et al. 2025 |
| *Vishniacozyma sinopodophylli* | CGMCC 2.6857^T^ | Leaf of *Sinopodophyllum hexandrum* | Tibet, China | OP470297 | OP470201 | OP853421 | OP771480 | OP853494 | Jiang et al. 2024 |
| *Vishniacozyma taibaiensis* | AS 2.2444^T^ | Plant leaves | Tabai mountains, China | AY557601 | AY557601 | LR814017 |  | LR814016 | Wang et al. 2011 |
| *Vishniacozyma taiwanica* | BCRC 23477^T^ | Leaves of *Avicennia marina* | Taiwan | MT906464 | MT906477 |  |  |  | Chang et al. 2021 |
| *Vishniacozyma tephrensis* | CBS 8935^T^ | Soil | Iceland | DQ000318 | DQ000318 | KF036407 | KF036821 | KF037092 | Vishniac 2002; Liu et al. 2015 |
| *Vishniacozyma terrae* | KCTC 27988^T^ | Soil | Pocheon City, Korea | MZ734447 | MZ734225 |  |  |  | Maeng et al. 2022 |
| *Vishniacozyma tianchiensis* | NYNU 236163^T^ | Phylloplane of *Salix matsudana* | Henan province, China | OR426458 | OR426457 |  |  |  | Liu et al. 2025 |
| *Vishniacozyma victoriae* | CBS 8685^T^ | Soil | Antarctica | AF444469 | AF363647 | KF036413 | KF036827 | KF037097 | Montes et al.1999; Liu et al. 2015 |
| *Vishniacozyma zhenxiongensis* | CGMCC 2.6901^T^ | *Pteridophyta* sp. | Yunnan province, China | OP470301 | OP470205 | OP784873 | OP771484 | OP853495 | Jiang et al. 2024 |
| *Tremella globispora* | CBS 6972^T^ | Basidiomata on *Diaporthe* sp. on pacific dogwood (*Cornus nuttalli*) | Canada | NR_155889 | NG_057771 | KF036531 | KF036947 | KF037208 | Findley et al. 2009 |
| *Hannaella sinensis* | CBS 7238^T^ | Leaf of *Triticum* sp. (wheat) | China | AF444468 | AF189884 | KF036483 | KF036897 | KF037160 | Scorzetti et al. 2002 |
