# Supplementary material for: Three new yeast species of Vishniacozyma (Bulleribasidiaceae, Tremellales) from different habitats
Source: MycoKeys. 2026 Feb 11;128:231–48. doi: 10.3897/mycokeys.128.175380 (PMC12917494; doi:10.3897/mycokeys.128.175380)
Supplement: Supplementary material 2 — Phenotypic properties of the novel Vishniacozyma species and phylogenetically closely related species [file mycokeys-128-231-s002.docx]

**Table S2.** Phenotypic properties of the novel *Vishniacozyma* species and phylogenetically closely related species.

| Substrate / Species | *Vishniacozyma pseudofoliicola* sp. nov***.*** | *Vishniacozyma foliicola ^a^* | *Vishniacozyma kombuchae* sp. nov. | *Vishniacozyma phoenicis ^b^* | *Vishniacozyma fructicola*  sp. nov*.* | *Vishniacozyma tephrensis ^c^* |
| --- | --- | --- | --- | --- | --- | --- |
| Glucose fermentation | - | - | - | - | - | - |
| Assimilation: |  |  |  |  |  |  |
| Glucose | + | + | + | + | + | + |
| Galactose | + | + | + | + | + | +, W |
| L-sorbose | - | - | S | W | W | +, W |
| Sucrose | + | + | + | + | + | +, W |
| Maltose | + | + | + | + | + | + |
| Cellobiose | + | + | + | + | + | + |
| Trehalose | + | + | + | + | + | +, W |
| Lactose | + | + | + | + | + | + |
| Melibiose | + | + | + | + | + | + |
| Raffinose | + | + | + | + | + | +, W |
| Melezitose | + | + | + | + | + | + |
| Inulin | - | W | - | - | - | +, W |
| Soluble starch | - | - | - | W | - | +, W |
| D-xylose | + | + | + | + | + | + |
| L-arabinose | + | + | + | + | + | + |
| D-arabinose | S, W | + | + | + | + | + |
| D-ribose | -, W | + | + | + | + | + |
| L-rhamnose | + | + | + | + | + | + |
| D-glucosamine | - | W | S | - | + | -,+ |
| Methanol | - | - | - | - | - | - |
| Ethanol | - | - | - | W | - | - |
| Glycerol | - | - | - | + | + | -, + |
| Erythritol | + | + | + | + | + | +, W |
| Ribitol | -, W | + | W | + | + | +, W |
| Galactitol | - | + | + | + | - | +, W |
| D-mannitol | - | + | + | + | + | - |
| D-glucitol | - | + | + | + | + | - |
| *myo*-Inositol | + | + | + | + | + | +, W |
| α-Methyl-D-glucoside | + | + | + | W | + | +, W |
| Salicin | - | + | W | + | + | +, W |
| DL-lactic acid | W | W | W | W | W | - |
| D-glucuronic acid | S, + | W | + | + | W | + |
| Succinic acid | S, + | W | - | + | + | -, W |
| Citric acid | S, W | + | - | + | - | - |
| 2-Keto-D-gluconate | + | nd | nd | nd | + | + |
| 5-Keto-D-gluconate | + | + | + | + | + | +, W |
| Arbutin | + | nd | W | + | + | +, W |
| Hexadecane | - | - | - | - | nd | nd |
| Nitrogen sources: |  |  |  |  |  |  |
| Nitrate | - | - | - | + | + | - |
| Nitrite | nd | + | nd | nd | nd | -, W |
| L-lysine | + | + | + | + | + | -, + |
| Cadaverine | nd | - | nd | nd | - | -, W |
| Ethylamine hydrochloride | - | + | - | nd | - | -, W |
| Creatine | - | - | - | + | + | nd |
| Creatinine | nd | nd | nd | + | + | - |
| D-glucosamine | + | + | + | + | + | nd |
| Growth at / in / with: |  |  |  |  |  |  |
| Growth at 28°C | W | + | + | + | + | nd |
| Growth at 29°C | - | + | + | + | + | nd |
| Growth at 30°C | - | - | + | + | - | - |
| 50% Glucose | S, + | - | -, W | + | + | - |
| 10% NaCl / 5% Glucose | -, W | nd | W | + | + | - |
| 16% NaCl / 5% Glucose | - | nd | - | nd | - | nd |
| 0.1% Cycloheximide | - | - | - | - | - | - |
| 0.01% Cycloheximide | W | - | W | W | W | -, W |
| Starch formation | + | + | + | + | + | +, W |
| Growth without vitamins | + | + | - | + | + | - |
| Urease | + | + | + | + | + | +, D |
| DBB | + | + | + | + | + | + |
|  |  |  |  |  |  |  |
| W – weak, D – delay, S – slow, nd – no data. | |  |  |  |  |  |
| ^a^ Wang et al. 2011 |  |  |  |  |  |  |
| ^b^ Crous et al. 2020 |  |  |  |  |  |  |
| ^с^ https://theyeasts.org/ (accessed on 5 August 2025) | |  |  |  |  |  |
